# Automated identification of protein-ligand interaction features using Inductive Logic Programming: a hexose binding case study

**DOI:** 10.1186/1471-2105-13-162

**Published:** 2012-07-11

**Authors:** Jose C A Santos, Houssam Nassif, David Page, Stephen H Muggleton, Michael J E Sternberg

**Affiliations:** 1 Computational Bioinformatics Laboratory, Department of Computer Science, Imperial College London, London, SW7 2BZ, UK; 2 Department of Computer Sciences, Department of Biostatistics and Medical Informatics, University of Wisconsin-Madison, Madison, WI-53706, USA; 3 Centre for Bioinformatics, Department of Life Sciences, Imperial College London, London, SW7 2AZ, UK

## Abstract

**Background:**

There is a need for automated methods to learn general features of the interactions of a ligand class with its diverse set of protein receptors. An appropriate machine learning approach is Inductive Logic Programming (ILP), which automatically generates comprehensible rules in addition to prediction. The development of ILP systems which can learn rules of the complexity required for studies on protein structure remains a challenge. In this work we use a new ILP system, ProGolem, and demonstrate its performance on learning features of hexose-protein interactions.

**Results:**

The rules induced by ProGolem detect interactions mediated by aromatics and by planar-polar residues, in addition to less common features such as the aromatic sandwich. The rules also reveal a previously unreported dependency for residues cys and leu. They also specify interactions involving aromatic and hydrogen bonding residues. This paper shows that Inductive Logic Programming implemented in ProGolem can derive rules giving structural features of protein/ligand interactions. Several of these rules are consistent with descriptions in the literature.

**Conclusions:**

In addition to confirming literature results, ProGolem’s model has a 10-fold cross-validated predictive accuracy that is superior, at the 95% confidence level, to another ILP system previously used to study protein/hexose interactions and is comparable with state-of-the-art statistical learners.

## Background

Elucidating unifying features of protein-ligand interactions in systems showing a diversity of interaction modes remains a challenging problem, often requiring extensive human intervention. In this work we present an automated general approach to identify these features using Inductive Logic Programming (ILP). We apply ILP to study the factors relevant to protein-hexose binding.

Hexoses are 6-carbon monosaccharides involved in numerous biochemical processes, including energy release and carbohydrate synthesis [1]. Several non-homologous protein families bind hexoses using a diverse set of protein-ligand interactions. Several research groups have used computational techniques to model and analyze hexose- and sugar-protein interactions, often employing extensive visualization and empirical methods [2-5]. Some techniques use surface and binding site similarities to search for matching functional sites in other proteins [6,7]. Others apply machine learning algorithms to construct sugar-specific classifiers [8,9]. Such classifiers can be combined with programs that detect protein surface-pockets of a given size [10,11] to discriminate potential binding-sites.

Recently Nassif et al. [12] used the ILP system, Aleph [13], to study hexose binding. A powerful feature of ILP is that, in addition to prediction, it automatically learns rules which can be readily understood. It has been successfully applied to predict and model various medical [14,15] and biological datasets [16,17]. However, the complexity and size of the hypothesis space often presents computational challenges in search time which limit both the insight and the predictive power of the rules found.

Recognizing the limitations of Aleph and other current ILP systems, Muggleton et al. [18] developed ProGolem to facilitate the learning of long, complex rules. Such rules are common in molecular biology and we propose that a sophisticated ILP system such as ProGolem is a promising approach to automatically learn these rules from molecular data.

The present work extends previous hexose prediction work in several ways. First we supplement the background knowledge with both atomic and amino-acid information. Second, we bias the hypothesis space to reduce the search space and increase the likelihood of generating meaningful rules. Third, we employ the newly-developed ProGolem, which has been shown to learn better than Aleph in highly non-determinate domains such as this hexose-binding application. Finally, we explore several approaches to curb the limitations of the *recall* bound, the maximum number of times a predicate may succeed, in ILP systems.

The combined usage of an extended background knowledge, a better biased search, and the ILP system ProGolem allowed the discovery of more accurate and insightful rules explaining the stereochemistry of hexose binding. Automatically finding these stereochemical rules and providing their explanation is the main contribution of this paper. While some of the rules ProGolem found were already known from the literature, other rules, namely one that specifies a dependency over residues cys and leu, have never been reported but are plausible and require further investigation.

Predicting whether an actual protein binds an hexose is of secondary importance to us. Nevertheless, the predictive accuracy of our approach is competitive to statistical learners such as Support Vector Machines and superior to the logic-based approach Aleph applied to study hexose/protein interactions [12].

### Dataset

For ease of comparison, we use the same dataset and cross-validation folds described in Nassif et al. [12]. They obtained the positive examples from the Protein Data Bank (PDB) [19] by selecting proteins with coordinates for bound ligands from the most common hexoses: galactose, glucose and mannose [20]. Theoretical structures and files older than PDB format 2.1 were ignored. Glycosylated sites and redundant structures (at most 30% overall sequence identity using PISCES [21]) were also ignored. The positive subset consisting of 80 protein-hexose binding sites (33 galactose, 35 glucose and 12 mannose) is presented in Table 1.

**Table 1:** The positive dataset, composed of 80 non-redundant protein-hexose binding sites

**Hexose**	**PDB ID**	**Ligand**	**PDB ID**	**Ligand**
Glucose	1BDG	GLC-501	1ISY	GLC-1471
	1EX1	GLC-617	1J0Y	GLC-1601
	1GJW	GLC-701	1JG9	GLC-2000
	1GWW	GLC-1371	1K1W	GLC-653
	1H5U	GLC-998	1KME	GLC-501
	1HIZ	GLC-1381	1MMU	GLC-1
	1HIZ	GLC-1382	1NF5	GLC-125
	1HKC	GLC-915	1NSZ	GLC-1400
	1HSJ	GLC-671	1PWB	GLC-405
	1HSJ	GLC-672	1Q33	GLC-400
	1I8A	GLC-189	1RYD	GLC-601
	1ISY	GLC-1461	1S5M	AGC-1001
	1SZ2	BGC-1001	1SZ2	BGC-2001
	1U2S	GLC-1	1UA4	GLC-1457
	1V2B	AGC-1203	1WOQ	GLC-290
	1Z8D	GLC-901	2BQP	GLC-337
	2BVW	GLC-602	2BVW	GLC-603
	2F2E	AGC-401		
Galactose	1AXZ	GLA-401	1MUQ	GAL-301
	1DIW	GAL-1400	1NS0	GAL-1400
	1DJR	GAL-1104	1NS2	GAL-1400
	1DZQ	GAL-502	1NS8	GAL-1400
	1EUU	GAL-2	1NSM	GAL-1400
	1ISZ	GAL-461	1NSU	GAL-1400
	1ISZ	GAL-471	1NSX	GAL-1400
	1JZ7	GAL-2001	1OKO	GLB-901
	1KWK	GAL-701	1OQL	GAL-265
	1L7K	GAL-500	1OQL	GAL-267
	1LTI	GAL-104	1PIE	GAL-1
	1R47	GAL-1101	1S5D	GAL-704
	1S5E	GAL-751	1S5F	GAL-104
	1SO0	GAL-500	1TLG	GAL-1
	1UAS	GAL-1501	1UGW	GAL-200
	1XC6	GAL-9011	1ZHJ	GAL-1
	2GAL	GAL-998		
Mannose	1BQP	MAN-402	1KZB	MAN-1501
	1KLF	MAN-1500	1KZC	MAN-1001
	1KX1	MAN-20	1KZE	MAN-1001
	1KZA	MAN-1001	1OP3	MAN-503
	1OUR	MAN-301	1QMO	MAN-302
	1U4J	MAN-1008	1U4J	MAN-1009

The table lists the protein’s PDB ID and the hexose ligand considered.

Their negative dataset consists of 80 PDB examples: 22 binding sites that bind hexose-like ligands (e.g. hexose or fructose derivatives, 6-carbon molecules, and molecules similar in shape to hexoses), 27 other-ligand binding sites and 31 non-binding sites. The non-binding sites are surface pockets that look like binding sites but are not known to bind any ligand. The negative dataset is presented in Table 2 (non-hexose binding sites) and Table 3 (non-binding sites).

**Table 2:** Protein binding-sites that bind non-hexose ligands

**PDB ID**	**Cavity center**	**Ligand**	**PDB ID**	**Cavity center**	**Ligand**
Hexose-like ligands					
1A8U	4320, 4323	BEZ-1	1AI7	6074, 6077	IPH-1
1AWB	4175, 4178	IPD-2	1DBN	pyranose ring	GAL-102
1EOB	3532, 3536	DHB-999	1F9G	5792, 5785, 5786	ASC-950
1G0H	4045, 4048	IPD-292	1JU4	4356, 4359	BEZ-1
1LBX	3941, 3944	IPD-295	1LBY	3944, 3939, 3941	F6P-295
1LIU	15441, 15436, 15438	FBP-580	1MOR	pyranose ring	G6P-609
1NCW	3406, 3409	BEZ-601	1P5D	pyranose ring	G1P-658
1T10	4366, 4361, 4363	F6P-1001	1U0F	pyranose ring	G6P-900
1UKB	2144, 2147	BEZ-1300	1X9I	pyranose ring	G6Q-600
1Y9G	4124, 4116, 4117	FRU-801	2B0C	pyranose ring	G1P-496
2B32	3941, 3944	IPH-401	4PBG	pyranose ring	BGP-469
Other ligands					
11AS	5132	ASN-1	11GS	1672, 1675	MES-3
1A0J	6985	BEN-246	1A42	2054, 2055	BZO-555
1A50	4939, 4940	FIP-270	1A53	2016, 2017	IGP-300
1AA1	4472, 4474	3PG-477	1AJN	6074, 6079	AAN-1
1AJS	3276, 3281	PLA-415	1AL8	2652	FMN-360
1B8A	7224	ATP-500	1BO5	7811	GOL-601
1BOB	2566	ACO-400	1D09	7246	PAL-1311
1EQY	3831	ATP-380	1IOL	2674, 2675	EST-400
1JTV	2136, 2137	TES-500	1KF6	16674, 16675	OAA-702
1RTK	3787, 3784	GBS-300	1TJ4	1947	SUC-1
1TVO	2857	FRZ-1001	1UK6	2142	PPI-1300
1W8N	4573, 4585	DAN-1649	1ZYU	1284, 1286	SKM-401
2D7S	3787	GLU-1008	2GAM	11955	NGA-502
3PCB	3421, 3424	3HB-550			

The table lists the protein’s PDB ID, the ligand considered and the specified cavity center. 22 ligands are similar to hexoses in shape and/or size. The cavity center is the centroid of the reported PDB atom numbers.

**Table 3:** Non-binding sites negative dataset, composed of random surface pockets that do not bind any ligand

**PDB ID**	**Cavity center**	**PDB ID**	**Cavity center**	**PDB ID**	**Cavity center**
1A04	1424, 2671	1A0I	1689, 799	1A22	2927
1AA7	579	1AF7	631, 1492	1AM2	1277
1ARO	154, 1663	1ATG	1751	1C3G	630, 888
1C3P	1089, 1576	1DXJ	867, 1498	1EVT	2149, 2229
1FI2	1493	1KLM	4373, 4113	1KWP	1212
1QZ7	3592, 2509	1YQZ	4458, 4269	1YVB	1546, 1814
1ZT9	1056, 1188	2A1K	2758, 3345	2AUP	2246
2BG9	14076, 8076	2C9Q	777	2CL3	123, 948
2DN2	749, 1006	2F1K	316, 642	2G50	26265, 31672
2G69	248, 378	2GRK	369, 380	2GSE	337, 10618
2GSH	6260				

The table lists the protein’s PDB ID and the specified cavity center, computed as the centroid of the reported PDB atom numbers.

The data also specify the center for each of the resulting 160 examples. The binding-site center is computed as the hexose pyranose ring centroid for the positive examples, and as the ligand or empty pocket centroid for the negative ones. The hexose pyranose-ring atoms are located up to 2.9 Å away from the ring’s centroid. Since some atomic interactions can be important up to 7 Å [22], we consider the binding-site as all protein atoms present within a 10 Å radius sphere around the binding center. All other atoms are discarded.

### Inductive logic programming

ILP is a machine learning approach that generates a hypothesis composed of a set of logical if-then rules that explains a given dataset [23]. ILP has three major advantages over other machine learning and data mining techniques. First, it allows an easy interaction between humans and computers by using background knowledge to guide the search. Second, it returns results in an easy-to-understand if-then format. Finally, ILP can easily operate on relational databases, as relational databases are naturally expressed as relations in first-order logic.

Most leading ILP approaches start by a saturation step, randomly selecting a positive example for which they construct the *bottom clause*: the most specific hypothesis that explains the example. This most specific clause is the rule formed by the conjunction of all features (called predicates or literals) pertaining to the chosen example. In the reduction step, ILP generalizes this rule (called clause or hypothesis) to include other positive examples using one of two basic induction methods, generalization or specialization.

Aleph, using a specialization approach, starts with the most general hypothesis, “all sites are hexose-binding sites”, calling all examples positives. It then refines this hypothesis by repeatedly adding the literal from the bottom-clause that best improves the hypothesis score. The new rule will be more specific, covering only a subset of the examples previously covered.

ProGolem, in contrast, uses a generalization search. Starting with the bottom clause, it successively drops a minimal set of literals to allow coverage of one additional positive example. By dropping this set of literals the clause becomes more general, and will cover a superset of the examples previously covered.

Both ProGolem and Aleph stop hypothesis refinement when the hypothesis score stops improving. A rule scores well if it covers many positive and few negative examples. If the rule passes a certain performance threshold, it is added to the *theory*, and all the positive examples it covers are removed. The cycle of saturation and reduction continues on the remaining examples. When all positive examples are covered or no new rules can be found, the ILP system outputs its theory, the set of the best rules found so far. Then, in the testing stage, a new instance is classified as positive (i.e. hexose-binding) if it is covered by any of the theory rules, otherwise it is labeled as negative.

The newly developed ProGolem is more than a specific-to-general version of Aleph. Two additional features set it apart. Aleph adopts a local theory construction method, incrementally adding a new rule to its theory after each reduction cycle. This method depends on the ordering of the positive examples, and it is possible that the best rules are not generated. This situation may occur if these better rules would be generated by examples that were removed by previous sub-optimal rules. By contrast, ProGolem implements a global theory construction approach, which ensures that the theory is only constructed after all rules have been generated. ProGolem repeatedly adds to the theory the rule that best improves the global theory score. The global-theory-construction feature of ProGolem is especially useful in this application.

The second feature makes ProGolem specifically suitable for our application. In ILP an example can have multiple instances from the same predicate. For example, a binding site has multiple atoms. If a predicate has more than one possible solution, it is called *non-determinate*. Hence the site *has_atom* predicate is non-determinate. Our hexose dataset is highly non-determinate.

When evaluating a clause, Aleph will proceed literal by literal from left to right. This is the standard Selective Linear Definite (SLD) resolution [24], which is the only option in most ILP systems. However, ProGolem has to evaluate longer clauses than Aleph due to its specific-to-general hypothesis search. SLD-resolution is too slow to compute the coverage of such long clauses. To cope with this problem ProGolem supports the usage of different resolution engines, including the smallest variable domain resolution, which enumerates the possible values a variable in a literal may take and, during clause evaluation, chooses at each moment the variable with the smallest domain [25]. This clause evaluation engine is better suited to our problem than SLD-resolution, drastically reducing the runtime per evaluated clause.

### Background knowledge

The background knowledge is the set of features, facts and rules known a priori. This is given to the ILP system as a basis for learning and constructing the classification rules. The piece of background knowledge central to our task is the binding site representation. Table ?? is an excerpt of the background knowledge for protein 1BDG. The *center_coords* predicate specifies the binding-site center coordinates, which is the pyranose ring centroid of the bound glucose in this structure. The *has_aminoacid* predicate specifies each amino acid present within the protein binding site, listing its unique identifier and name. The *has_atom* predicate details the residue atoms, specifying the PDB atom name and its coordinates. By extracting the coordinates of the center and the various atoms, we compute their respective distances. We set a tolerance of 0.5 Å on distances between atoms, a sensible error margin in a hexose binding site [26].

In addition to these facts, ILP allows for a higher level of expressiveness within its background knowledge: human coded rules. Using the facts of Table 4, and the Euclidean distances between atoms that are derived from this data, we can now define the predicates *atom_to_center_dist* and *atom_to_atom_dist*. These predicates respectively compute the distance between an atom and the binding-site center, and between two atoms. We also define a *diff_aminoacid* predicate which allows expressing that two amino acids are different. This may be relevant when there are multiple amino acids of the same type and each amino acid needs to be individually identified.

**Table 4:** Excerpt of the background knowledge for protein 1BDG in Prolog

center_coords(p1BDG, p(27.0,22.1,64.9)).
has_aminoacid(p1BDG, a64, phe).
has_aminoacid(p1BDG, a85, leu).
has_aminoacid(p1BDG, a86, gly).
has_aminoacid(p1BDG, a87, gly).
has_atom(p1BDG, a64, ’CD2’, p(22.4,13.3,65.5)).
has_atom(p1BDG, a64, ’CE2’, p(21.6,14.0,66.4)).
has_atom(p1BDG, a85, ’C’, p(24.6,25.9,57.4)).
has_atom(p1BDG, a85, ’O’, p(24.6,24.8,57.8)).
has_atom(p1BDG, a86, ’N’, p(24.8,27.0,58.3)).
has_atom(p1BDG, a86, ’CA’, p(24.9,26.8,59.7)).

Since 1BDG is a hexose-binding protein, center_coords/2 predicate states the coordinates of the hexose binding center. The has_aminoacid and has_atom predicates state the coordinates of the amino acids and atoms in a neighborhood of 10 Å of the binding site center.

### Hypothesis space

We experiment with multiple hypothesis spaces. Similarly to Nassif et al. [12], we first exclude residue information and limit the background to the atoms and their 3D coordinates. In this *atom-only* representation, the binding site is a sphere of radius 10 Å containing atoms in space, for which distances can be computed (see Table 5).

**Table 5:** Background knowledge predicates for the two binding site representations

**Representation**	**Background knowledge predicates**
atom-only	center_coords/2, has_atom/4, dist/4
amino acid	has_aminoacid/3, atom_to_center_dist/4,
	atom_to_atom_dist/6, diff_aminoacid/2

The /N indicates the arity of the background knowledge predicate. For instance, given a binding site, the center_coords predicate returns the coordinates of its center (1 input + 1 output arguments = 2).

The distances between atoms are computed by having a *dist* literal in the ILP background knowledge, allowing ILP systems to express the 3D conformation of the binding site. However, the number of possible distances grows quadratically with the number of atoms considered, resulting in an exponential growth of the bottom clause. Starting its generalization search from the bottom clause, ProGolem learning time is highly sensitive to its length. To keep learning tractable, both ProGolem and to a lesser extent Aleph, require a bound on the maximum number of solutions a given predicate may return, called the *recall* (not to be confused with the statistical measure of the same name, also called sensitivity). In practice this *recall* bound limits the hypothesis search space by forcing that only the **first***recall* solutions of a literal be considered in the bottom clause.

By relying on the ordering of the atoms and residues in the background knowledge, having a bound on the *recall* of a predicate is an approach subject to data idiosyncrasies as the ordering of the background knowledge predicates is arbitrary and only the first *recall* are considered. In this work we explore two alternative approaches of organizing the background knowledge to curb the limitations of having a recall bound. The first approach, *randomized recall*, considers all solutions first, out of which it randomly picks a number equal to *recall*; rather than the first *recall* atoms in the binding-site data representation. This is achieved by either altering the internal recall routine, as we did, or equivalently, by randomizing the order of the atoms and residues in the background knowledge.

The second approach is *domain-dependent*. Using Random Forests to measure feature importance [27,28], Nassif et al. [9] show that atoms closest to the binding center have higher discriminative power. Closest atoms are more likely to determine whether or not the binding site binds hexose, as compared to more distant atoms. The *domain-dependent* approach orders the atoms and residues in the background knowledge by their distance from the binding site center. For instance, in this approach, the distance literal will attempt to match the *recall* atoms closest to the binding center.

Another contribution of this work is the re-modeling of the problem representation and a better bias to the hypothesis space. We propose two major improvements to the atomic representation. First is the inclusion of residues using the *has_aminoacid* predicate. The second is imposing that atoms cannot appear dangling in a hypothesis. A residue has to be introduced into a rule first, before *atom_to_atom_dist* and *atom_to_center_dist* predicates compute its atomic distances. We thus only compute distances between atoms of residues already in a rule. In this *amino acid* representation, the binding site sphere is composed of amino acids, which in turn contain atoms (Table 4).

By first dealing with residues instead of atoms, the binding site sphere now contains a smaller number of elements. In addition, in the *amino acid* representation we can express the distance between two atoms using only one literal, *atom_to_atom_dist*. A rule can contain up to *recall* residues, and for each atom of a given residue we measure its distance to *recall* atoms of each one of the included residues. In contrast, in the *atom-only* representation we need three literals to express a distance, two *has_atom* and one *dist*. A rule can contain only *recall* atoms, and each atom can only detect *recall* other atoms in the feature space. Thus, for the same recall bound, the *amino acid* representation considers both more features and generates more informative clauses than the *atom-only* representation.

Table 6 is an example of an *amino acid* representation hypothesis, in raw Prolog format as induced by ProGolem. A Prolog clause follows a *Head:-Body* structure. The head is verified (i.e. is true) if the body (a conjunction of literals) holds true. The uppercase letters in the clause, in this case A, B, C and D, are logical variables and represent a certain entity. Lowercase strings, string within quotes (e.g. leu, cys, ’N’, ’OD2’, and ’C’) and numbers are constants representing themselves. The variable A in this clause represents a protein, variables B, C and D represent amino acids.

**Table 6:** An *amino acid* representation hypothesis

bind(A):-	
	has_aminoacid(A,B,asp),
	atom_to_atom_dist(B,B,’N’,’OD2’,4.6,0.5),
	has_aminoacid(A,C,leu),
	has_aminoacid(A,D,cys),
	atom_to_center_dist(B,’C’,7.6,0.5).

English translation: A protein is hexosebinding if the N and OD2 atoms of an aspartic acid are 4.6+/-0.5 Angstroms away from each other and the C atom of this aspartic acid is 7.6+/-0.5 Angstroms away from the binding center, a leucine and a cysteine are also present.

## Methods

All materials (i.e. dataset, ILP systems and scripts) to reproduce these experiments are available at http://www.doc.ic.ac.uk/∖∼jcs06/Hexose.

### ILP settings

We apply two ILP systems, Aleph and ProGolem, with both *atom-only* and *amino acid* representations, and use YAP 6.0.6 as the Prolog compiler [29]. To ensure a fair comparison, we use the same settings for both ILP algorithms whenever possible. We set the recall bound to 7, and the maximum number of negatives a hypothesis may cover to 5. We evaluate clauses according to the usual scoring function in ILP, compression: *positive examples covered - negative examples covered - clause length*. For instance, for the same clause length, say 6 literals, a clause covering 37 positives and 4 negatives has a better score (37−4−6=27) than one covering 30 positives and no negatives (30−0−6=24).

We use ProGolem with its global theory construction and smallest variable domain resolution. In Aleph, we set the number of nodes to be explored when searching for an acceptable clause to 5000. The clause length in Aleph, i.e. the maximum number of literals allowed in a hypothesis, was set to match the clause length that ProGolem generates (5 for *atom-only*, 6 for *amino acid*). If the same clause length was used in both representations, the predictive accuracies of Aleph would be lower. In ProGolem, the user does not need to specify the maximum clause length of a rule, as the hypothesis search is from specific to general.

### Homology and cross-validation

Our dataset consists of 160 binding sites, belonging to 152 unique proteins (8 of the hexose-binding proteins have two distinct binding sites). These 152 proteins belong to a total of 122 CATH [30] superfamilies. In order to guarantee that rules are not being learned from homologous proteins, the correct procedure is that each cross-validation fold does not contain proteins whose superfamilies (i.e. homologues) also occur in other folds.

Unfortunately, with this particular dataset, it is impossible to construct cross-validation folds that verify this non-sharing superfamily constraint. This is because the binding site may span multiple chains, each belonging to a different superfamily. Moreover, a single chain may be subdivided into domains, each belonging to different CATH superfamilies. Thus, if binding site *A* belongs to superfamilies *sf*1 and *sf*2, *B* to *sf*2 and *sf*3, and *C* to *sf*3 and *sf*4, the binding sites *A*, *B* and *C* must be in the same cross-validation fold. With our dataset, this constraint would result in a single cross-validation fold containing 48 binding sites (34 positives, 14 negatives) out of 160, creating a significant imbalance between folds.

Given this impossibility, and in order for our results to be comparable with previous work, we performed a 10-fold cross-validation using the same folds as [12]. Since the number of hexose binding proteins is limited, the dataset proteins share a low sequence identity (≤30*%*), and the main goal of this paper is to provide insight into the hexose-binding discriminating process rather than the predictive accuracy of the classifiers, we consider our methodology acceptable. Each fold consists of 8 positive and 8 negative examples. Following standard machine learning technique, where for small datasets a large number of folds is needed, we train ProGolem on 9 folds (144 examples) and test on the remaining fold (16 examples), repeating this process 10 times such that each fold is used for testing once [31]. We use the testing fold results to compute the relevant statistics. When comparing two approaches or algorithms on the 10 folds, we consistently use a two-tailed paired t-test at the 95% confidence level.

## Results and discussion

It is important to note that the main aim of this work is to discover rules describing the stereo-chemistry of protein-hexose binding. Although there is empirical evidence suggesting that many hexose dockings are not accompanied by substantial protein conformational changes [26], we do not aim to predict the binding sites of new hexose-binding proteins, as we would not know in advance the coordinates of the hexose ligand. Nevertheless, we use 10-folds cross-validated predictive accuracies as a measure to demonstrate the robustness of the rules.

### Altering ProGolem recall

Simply relying on the given order of the background knowledge introduces placement bias. Both randomizing recall selection, and incorporating domain knowledge by ordering the atoms according to their distance to the binding site center, significantly improves accuracy when compared to the given PDB ordering (*p*-values of 0.026 and 0.021, respectively). This showcases the importance of domain knowledge, whereas clever manipulations based on prior knowledge will have better results compared to default settings. We also argue that randomizing recall selection should be used as default since it avoids data idiosyncrasies.

As explained previously, an important parameter when running ILP systems, is the *recall* bound, which imposes a bound on the maximum number of solutions a given Prolog predicate may return. Since, for performance reasons, the *recall* setting has to be limited to a relatively low value, we started by performing experiments to determine how to best order the atom and residues to get the most out of a limited recall.

We considered three schemes. The first considers the atoms of the protein according to the order of their occurrence in the PDB file, which follows the order of the primary sequence. The second scheme randomizes the order of the atoms in the background knowledge. The third scheme, *domain-dependent*, orders the atoms by their distance to the binding-site center.

Using the *atom-only* representation, the three approaches respectively yield an accuracy of 59.4*%*, 68.8*%* and 74.4*%*(Table 7). Sorting the binding-site atoms according to their distance from the binding center outperforms randomizing them, which in turn outperforms using their PDB sequence order. We therefore adopt the *domain-dependent* approach to organize the atoms and residues in the background knowledge in all our subsequent runs, involving both ILP algorithms (ProGolem and Aleph) and both data representations (*atom-only* and *amino acid*).

**Table 7:** *Atom-only* representation 10-folds cross-validation predictive accuracies for ProGolem using different recall selection methods

	**Recall selection method**		
**Fold**	**Primary sequence**	**Randomized**	**Domain-dependent**
1	43.8*%*	56.3*%*	87.5*%*
2	62.5*%*	93.8*%*	78.5*%*
3	81.3*%*	87.5*%*	87.5*%*
4	56.3*%*	50.0*%*	43.8*%*
5	68.8*%*	68.8*%*	81.3*%*
6	37.5*%*	56.3*%*	81.3*%*
7	56.3*%*	62.5*%*	75.0*%*
8	68.8*%*	68.8*%*	81.3*%*
9	62.5*%*	81.3*%*	62.5*%*
10	56.3*%*	62.5*%*	68.8*%*
Mean	59.4*%*	68.8*%*	74.8*%*
Std Dev	12.6*%*	14.4*%*	13.4*%*

### ProGolem performance

Table 8 shows the 10-fold cross-validation predictive accuracies for Aleph and ProGolem with the *atom-only* and *amino acid* representations. We also compare our results to a state-of-the-art approach, which uses Random Forests [27] for feature selection, and Support Vector Machines (SVM) [32] for classification. Internal validation selects the best Random Forests and SVM parameters for each training fold before predicting the testing fold. Note that SVM is a statistical classifier requiring a constant-length feature vector as input. This necessitates a different problem representation than the one used with ILP. Essentially we divide the binding site in concentric spherical layers, and for each we compute atomic and residue properties. We also add various atomic features namely hydrophobicity, charge and hydrogen-bonding. Refer to Nassif et al. [9] for method and representation details.

**Table 8:** 10-folds cross-validation predictive accuracies for Aleph, ProGolem and SVM

	**Learning algorithm**				
**Fold**	**Aleph 1**	**ProG. 1**	**Aleph 2**	**ProG. 2**	**SVM**
1	50.0*%*	75.0*%*	56.3*%*	75.0*%*	81.3*%*
2	68.8*%*	81.3*%*	68.8*%*	81.3*%*	87.5*%*
3	62.5*%*	68.8*%*	68.8*%*	93.8*%*	87.5*%*
4	50.0*%*	56.3*%*	68.8*%*	75.0*%*	75.0*%*
5	75.0*%*	81.3*%*	56.3*%*	81.3*%*	75.0*%*
6	68.8*%*	87.5*%*	81.3*%*	87.5*%*	87.5*%*
7	75.0*%*	81.3*%*	75.0*%*	81.3*%*	93.8*%*
8	93.8*%*	81.3*%*	75.0*%*	93.8*%*	87.5*%*
9	68.8*%*	75.0*%*	75.0*%*	81.3*%*	75.0*%*
10	56.3*%*	56.3*%*	87.5*%*	81.3*%*	62.5*%*
Mean	66.9*%*	74.4*%*	71.3*%*	83.2*%*	81.3*%*
Std Dev	13.2*%*	10.8*%*	9.8*%*	6.6*%*	9.3*%*

The *1* besides Aleph and ProGolem stands for the *atom-only* representation and the *2* for the *amino acid* representation. SVM uses a different representation (see text).

From Table 8 we notice that ProGolem performs better using the enhanced *amino acid* representation rather than *atom-only* (*p*-value =0.029). However, the *amino acid* representation yields no statistically significant improvement in Aleph (*p*-value =0.39). A possible explanation as to why ProGolem takes advantage of the amino acid representation more than Aleph is the myopia effect [33]. The myopia effect occurs because general-to-specific ILP systems, like Aleph, indirectly assume literals are conditionally independent given the target class. They refine the working hypothesis by adding one literal at a time, the one that maximizes a fitness function. If literals have a strong conditional dependency, any selected literal will roughly have the same score. Thus multiple literals need to be added before Aleph can determine which set is optimal. If the literals are highly non-determinate, as is our case, a significant portion of the search resources is wasted searching very similar hypotheses, which results in a poorer chance of finding good theories.

ProGolem outperforms Aleph for both representations (Table 8). The differences in their predictive accuracies are statistically significant for both *atom-only* (*p*-value =0.043) and *amino acid* (*p*-value =0.004) representations, the latter being significant even at the 99% confidence level. This discrepancy is in part explained by ProGolem’s global theory construction, which only constructs the final theory after all hypotheses have been generated rather than incrementally, on a per-example basis, as Aleph does.

Finally, we compare ILP to SVM. Despite *amino acid* ProGolem having a higher average accuracy and a lower standard deviation than SVM, the difference is not statistically significant (*p*-value =0.52). More surprisingly, SVM does not significantly outperform *amino acid* Aleph (*p*-value =0.057). SVM significantly outperforms both Aleph (*p*-value = 0.005) and ProGolem (*p*-value = 0.025) in the *atom-only* representation.

### Insight from rules

In this section we present the English translation and the biological explanation for some of the most relevant rules found by ProGolem using the *amino acid* representation. Although these particular rules were generated from the whole data set, the rules found on each cross-validation fold are similar; the themes associated to hexose-binding are consistently identified by ProGolem on each fold. The actual positive and negative examples covered by each rule are presented in Table 9.

**Table 9:** Positive and negative examples covered by each reported ProGolem rule

**Rule**	**Positive examples**	**Negative examples**
	37: 1BDG, 1BQP, 1DZQ, 1HKC, 1HSJ_2, 1ISY, 1ISZ, 1ISZ_2,	
	1J0Y, 1JG9, 1JZ7, 1KLF, 1MMU, 1MUQ, 1NSU, 1NSX, 1NSZ,	
1	1OKO, 1OP3, 1OQL, 1OQL_2, 1OUR, 1PIE, 1Q33, 1S5M, 1SZ2,	4: 1AWB, 1W8N, 2B0C, 2B32
	1SZ2_2, 1TLG, 1U2S, 1U4J, 1U4J_2, 1UA4, 1UAS, 1WOQ,	
	2BQP, 2BVW, 2BVW_2	
	24: 1DJR, 1EUU, 1HIZ, 1HSJ, 1HSJ_2, 1KWK, 1KX1, 1KZA,	
2	1KZB, 1KZC, 1KZE, 1L7K, 1MUQ, 1NS8, 1NSM, 1NSU, 1NSX,	0
	1NSZ, 1PWB, 1S5D, 1S5E, 1SO0, 1TLG, 1XC6	
3	30: 1DIW, 1DJR, 1EUU, 1HIZ, 1ISZ, 1KX1, 1KZA, 1KZB, 1KZC,	0
	1KZE, 1L7K, 1LTI, 1NS0, 1NS2, 1NS8, 1NSM, 1NSU, 1NSX,	
	1NSZ, 1OKO, 1OQL_2, 1OUR, 1PWB, 1QMO, 1S5D, 1S5E, 1S5F,	
	1SO0, 1U2S, 2GAL	
4	7: 1HSJ, 1HSJ_2, 1KME, 1RYD, 1S5M, 1TLG, 1UGW	0
5	6: 1HIZ_2, 1KWK, 1QMO, 1U4J, 1U4J_2, 1XC6	0
	18: 1ISY, 1ISY_2, 1ISZ, 1ISZ_2, 1NF5, 1OKO, 1OQL, 1PIE,	
6	1R47, 1SO0, 1SZ2, p1SZ2_2, 1TLG, 1U4J, 1U4J_2, 1UAS,	0
	2BVW, 2BVW_2	

According to ProGolem, a site is hexose-binding if: 

1. It contains two different asn residues and an asp residue whose cg atom is 5.4±0.5 Å away from the binding center.[Positives covered = 37, Negatives covered = 4]

2. It contains an asn whose n and c atoms are 2.4±0.5 Å apart, and a glu whose cb and cg atoms are 8.0±0.5 Å and 6.9±0.5 Å away from the binding center, respectively.[Positives covered = 24, Negatives covered = 0]

3. It contains an asn residue whose n atom is 8.2±0.5 Å away from the binding center, and an asn residue whose n and nd2 atoms are 4.1±0.5 Å apart and whose n and o atoms are 3.6±0.5 Å apart.[Positives covered = 30, Negatives covered = 0]

4. It contains a trp residue whose cb atom is 7.1±0.5 Å away from the binding center, and whose n and cd1 atoms are 4.0±0.5 Å apart.[Positives covered = 14, Negatives covered = 0]

5. It contains a tyr residue whose cb and oh atoms are 5.6±0.5 Å apart, a his residue whose nd1 atom is 8.9±0.5 Å away from the binding center, and a tyr residue whose o atom is 9.8±0.5 Å away from the binding center.[Positives covered = 6, Negatives covered = 0]

6. It contains cys and leu residues, and an asp residue whose n and od2 atoms are 4.6±0.5 Å apart, and whose c atom is 7.6±0.5 Å away from the binding center.[Positives covered = 18, Negatives covered = 0]

Note that the binding center is the hexose pyranose ring centroid, and that up to the first 4 Å of the distance between a binding-site atom and the binding center are occupied by the docked hexose. In addition, hydrogen atoms are generally not included in PDB files. Thus the presence of an atom may be a surrogate for its hydrogen involved in a hydrogen-bond.

The first rule requires the presence of an asp and two asns. Previously, Rao et al. [34] highlighted the importance of both residues in hexose binding. Studying the lectin protein super-family, they report that the 3D positions of binding-site asp and asn residues are conserved. This holds despite lectins binding various types of hexoses and exhibiting different sugar-binding specificities.

That same rule requires the aspcg atom to be 5.4 Å away from the centroid of the hexose pyranose ring. The pyranose radius itself being 3 Å, the asp actually interfaces the docked hexose. Binding-site interface residues are key for hexose recognition and binding [9], especially planar polar residues that establish a network of hydrogen bonds with the various hydroxyl groups of the docked hexose [35]. Quiocho and Vyas [36] report that the most common planar polar amino acids involved in hexose binding are mainly asp and asn, followed by glu. ProGolem detects the role of glu in the second rule.

The second rule also implies a specific conformation with a triangular distance between glu’s cb and cg atoms, and the binding center. Sujatha and Balaji [26] report that spatial disposition of protein-galactose interacting atoms is not conserved *per se*, but is conserved with respect to the docking position of the ligand. Similarly, ProGolem often specifies the distance of an atom with respect to the centroid of the hexose.

**Figure 1:**
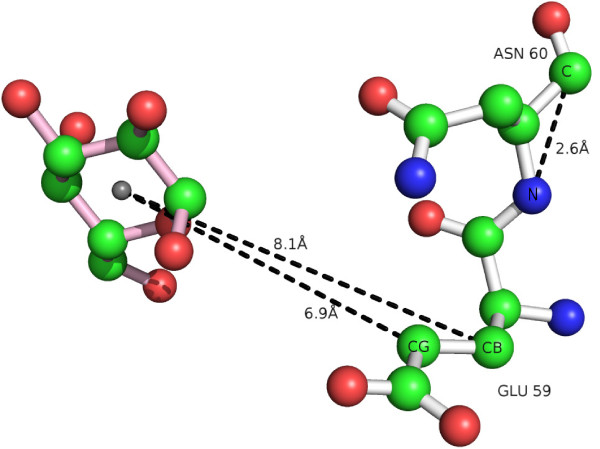
**Visualization of the second ProGolem rule instantiated with protein 1HIZ (covered by the rule).** The hexose is the glucose molecule to the left, with a pink backbone. To the right, with a white backbone, are the amino acids asn and glu, in closer contact with the hexose. The dotted black lines highlight the distances between the atoms in the amino acids and the center of the hexose.

An additional advantage of inducing rules using ILP is the straightforward reverse-engineering to find the particular proteins, residues and atoms covered by a given rule. This is achieved by executing the ILP rule in a Prolog interpreter. As an example, Figure 1 visualizes the second rule with protein 1HIZ, a xylanase. The hexose ligand is depicted with its backbone in light pink. The two amino acids involved in the rule, a glutamic acid and an asparagine, have a white backbone. The relevant distances are shown.

In addition to specifying the distance from the binding center, ProGolem can detect specific amino acid stereochemical dispositions. The third rule determines a particular asn conformation, specifying the distances between backbone n and o atoms, and the side chain nd2 atom. The various spatial dispositions of the different rules need further investigation to compare them with known 3D hexose binding-site conformations.

The aromatic residues (trp most frequently, tyr, phe, and to a lesser extent his) provide a stacking platform for the hexose to dock on [36]. The hexose pyranose ring forms a planar apolar hydrophobic side that stacks, through hydrophobic and van der Waals interactions, over the aromatic residues planar apolar hydrophobic side chain ring [37]. Similarly, the ProGolem fourth rule requires the presence of trp in a particular stereochemical conformation.

The fifth rule requires the presence of one or two tyr, and a his. This rule is thus describing a conformational representation of two or three aromatic residues around the binding-site center. It is interesting that this low-coverage rule may indeed be capturing the infrequent sandwich interaction, whereby two or more aromatic residues engage both faces of a hexose pyranose ring [38].

The last rule specifies cys and leu residues. Both have negative interface propensity measures (see below) and do not form hydrogen bonds with hexoses [39]. To quantify the disposition amino acids to be in contact with the docked sugar, Taroni et al. [39] devised an interface propensity measure, defined as the logarithm of the ratio between a surface residue frequency at the sugar binding site, and the average frequency of any surface residue at the binding site. They compute and report the sugar-interface propensity measure for the 20 common amino acids. A residue with a negative propensity measure does not favor the sugar binding-site region since it is present there less frequently than average.

This rule covers 18 positive examples and no negative examples, and clearly specifies the presence of cys and leu as a discriminative factor for hexose-binding site recognition. This dependency over leu and cys is not previously identified in literature and merits further attention.

## Conclusion

Inductive Logic Programming (ILP) is a leading technique to mine accurate and comprehensible rules. The newly developed ILP system ProGolem is well suited for complex non-determinate problems as often found in biological datasets. In our hexose-binding application, its predictive accuracy is significantly better than previous approaches, while showing a clear insight of the underlying discrimination process.

ProGolem was able to infer different aspects of the established biochemical information about hexose-binding, namely the presence of a docking aromatic residue, the importance of interface atoms, and the hydrogen-bonding activity of planar-polar residues (asn, asp, glu). ProGolem also detected the less common aromatic sandwich interaction.

In addition, ProGolem reveals an important previously unreported finding: a dependency over residues cys and leu. It also specifies stereo configurations involving aromatic and hydrogen bonding residues. The newly reported relationship and 3D conformations require further investigation.

Finally, we recommend randomizing the recall selection by default and have implemented this option in ProGolem. We also note that incorporating domain-dependent knowledge in parameter settings is likely to lead to the best results.

## Competing interests

Professors Muggleton and Sternberg are Directors and Shareholders in Equinox Pharma Ltd, a company which commercializes Inductive Logic Programming in the areas of bioinformatics and chemoinformatics. The remainder authors have no competing interests.

## Authors’ contributions

JCAS developed the ProGolem ILP system under the supervision of SHM. ProGolem’s theoretical foundations were laid out by SHM. HN created the hexose dataset and performed the Support Vector Machines evaluation. HN and Page did the initial experiments with Aleph. JCAS undertook the current experimental evaluation between ProGolem and Aleph. JCAS, HN and MJES wrote the paper. All authors read and approved the final manuscript.
